# Exploring wear at the nanoscale with circular mode atomic force microscopy

**DOI:** 10.3762/bjnano.8.266

**Published:** 2017-12-11

**Authors:** Olivier Noel, Aleksandar Vencl, Pierre-Emmanuel Mazeran

**Affiliations:** 1 IMMM, UMR CNRS 6283, Le Mans Université, Av. O. Messiaen, 72085 cedex 09, Le Mans, France; 2 Faculty of Mechanical Engineering, University of Belgrade, Kraljice Marije 16, 11120 Belgrade 35, Serbia; 3 Sorbonne universités, Université de Technologie de Compiègne, UMR CNRS 7337, Roberval, Centre de recherche de Royallieu – CS 60 319 – 60 203 Compiègne cedex, France

**Keywords:** circular mode atomic force microscopy, composite materials, image processing, nanowear, wear mechanisms

## Abstract

The development of atomic force microscopy (AFM) has allowed wear mechanisms to be investigated at the nanometer scale by means of a single asperity contact generated by an AFM tip and an interacting surface. However, the low wear rate at the nanoscale and the thermal drift require fastidious quantitative measurements of the wear volume for determining wear laws. In this paper, we describe a new, effective, experimental methodology based on circular mode AFM, which generates high frequency, circular displacements of the contact. Under such conditions, the wear rate is significant and the drift of the piezoelectric actuator is limited. As a result, well-defined wear tracks are generated and an accurate computation of the wear volume is possible. Finally, we describe the advantages of this method and we report a relevant application example addressing a Cu/Al_2_O_3_ nanocomposite material used in industrial applications.

## Introduction

Wear remains a prominent economical issue [1–5] as it generates industrial maintenance and limits the lifetime of numerous mechanical systems. Another major concern is the ecological impact of wear. For instance, wear is responsible for the production of microparticles from the abrasion of roads, brakes or car engines [6]. The use of lubricants to reduce wear is also a source of pollution as they are often in the form of unfriendly environmental chemicals discarded into the environment [7].

Advances in tribology have allowed for a better understanding of wear mechanisms at the macroscale [8–9]. In particular, different wear mechanisms such as abrasion, adhesion, surface fatigue, and tribochemical reactions [10] are known to depend on the tribological systems and the operating conditions. For example, the straightforward Archard’s law predicts that the wear volume is proportional to the normal load and the sliding distance and the inverse of the hardness of the material [11].

Exploring wear at the nanoscale becomes more and more mandatory with the development of the nanotechnology and its applications [12]. In addition, emerging numerical simulations of nanowear mechanisms are quite demanding in experimental validations [13–14]. The development of atomic force microscopy (AFM) in the 90’s has opened the field of tribology at the nanoscale. One of the main advantages of AFM is that a single asperity contact between a nanometer-sized AFM tip and an interacting surface can be generated [15–16]. Although AFM is used as a versatile technique, it was first dedicated to imaging and not to the measurement of tribological properties. Classical wear experiments with the AFM hinge on the slow linear scanning of the sample surface with the AFM tip with a constant normal load. Under such conditions, producing a significant wear is long and fastidious due to the low sliding velocity. In addition, the typical AFM scanning velocity, in the µm/s range, does not allow well-defined wear tracks to be obtained as the piezoelectric actuator thermal drift continuously moves the sample under the probe. As a significant impediment, one ends up with mostly large and low-depth wear tracks for which the wear volume is difficult to compute. In this case, the determination of relevant wear laws is difficult or even impossible. Finally, only a few proper experimental studies of nanoscale studies of wear (nanowear) have been reported in the literature [17–20], whereas AFM has been widely used for measuring nanoscale friction [21–22].

In this paper, we propose a new experimental methodology based on circular mode AFM (CM-AFM) to explore wear mechanisms and laws at the nanoscale. It consists of wearing the material with the AFM tip with a suitable circular motion at a high frequency. Following this process, a well-defined wear track obtained within a reasonable time may be easily revealed by AFM imaging. We also describe an original wear track image treatment that allows the wear volume and its uncertainties to be computed. Finally, an application of this methodology and computation for exploring wear of a specific copper-based composite with alumina nanoparticles is reported and discussed.

## Results and Discussion

### The CM-AFM: a powerful nanotribometer for wear experiments

**Figure 1:**
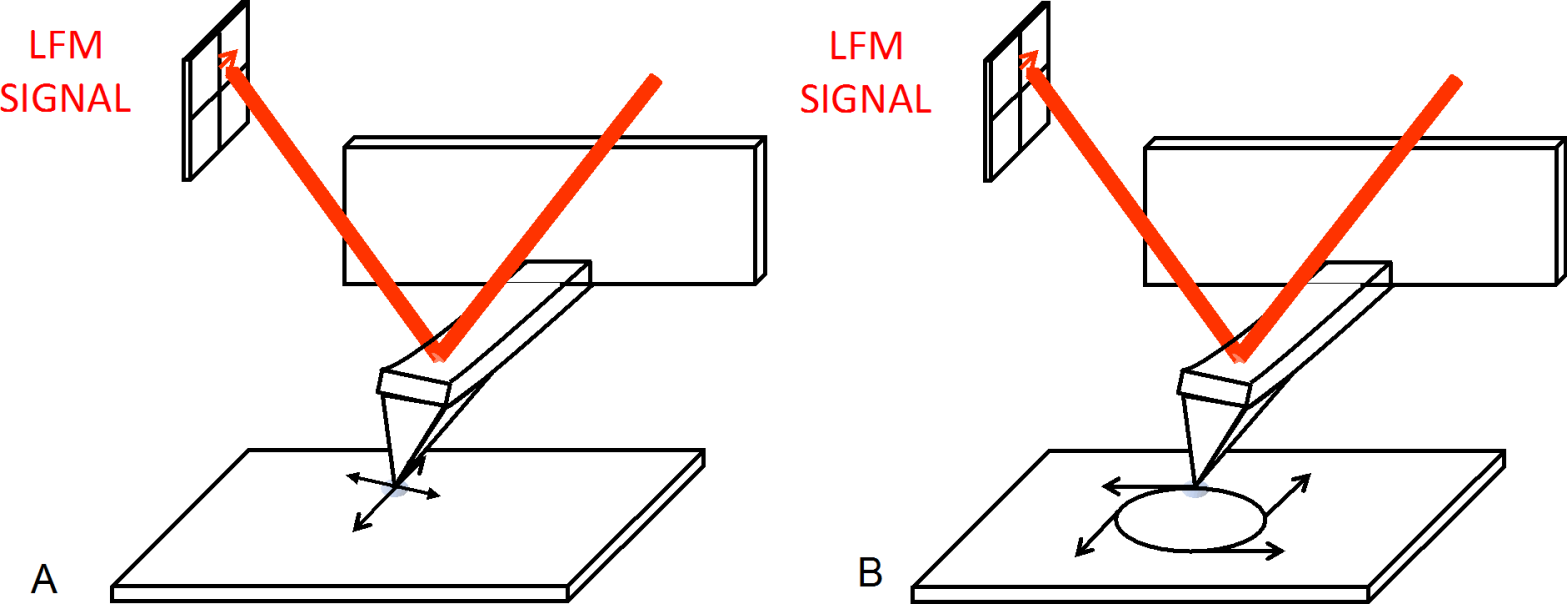
Schematic of the wear-induced atomic force microscopy (AFM) experimental protocols using (A) conventional scanning and (B) circular mode AFM circular displacement.

In classical wear experiments based on AFM, the probe linearly scans back and forth along the surface of the material while a constant normal load is applied on the contact (Figure 1A). With this problematic methodology, stop periods generated during the scan and a slow, nonconstant sliding velocity prohibit performing the experiments in a stationary regime. Recently, a new AFM mode called the CM-AFM [23–26] was implemented to overcome some of the limitations of the conventional wear experimental set-up using an AFM. In CM-AFM, the electronic driving unit of a conventional AFM has been modified to impose a relative circular displacement of an AFM tip in contact with the plane of a given material (Figure 1B). The circular displacement is provided by way of injecting two sinusoidal voltage components in phase quadrature on the piezoelectric actuator of the AFM in both directions of the plan of the sample.

**Figure 2:**
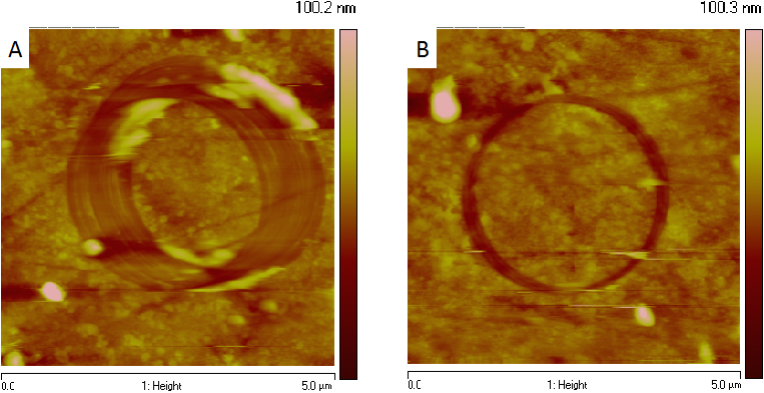
Atomic force microscopy (AFM) contact mode topographic images of wear tracks resulting from wear experiments on copper-based composites (the counter-body is a silicon nitride probe) at a constant normal load of 180 nN with constant sliding distance and sliding velocities of (A) 100 µm/s and (B) 1 mm/s. To maintain a constant sliding distance, the wear duration is 100 min for the experiment in (A) and 10 min for the experiment in (B). It is clearly demonstrated that a long wear time results in a large and low-depth wear track due to the drift.

With such a driving scheme, the sliding velocity of the nanocontact is constant and continuous. Stop periods do not occur during the experiment and real stationary regimes are successfully achieved. In addition, with CM-AFM, the sliding velocity is driven by the frequency and the amplitude of external sinusoidal voltages applied to the scanner. Therefore, wear experiments may be achieved at high sliding velocities (>6 mm/s) as compared to sliding velocity values that are implemented in classical AFM wear experiments (100 µm/s maximum). The only limitation for high sliding velocities is the resonance frequency of the scanner and its lateral extension. For example, for a conventional AFM scanner whose resonance frequency is about 500 Hz and considering a 4 µm diameter circular displacement (which is obtained by applying an external voltage of 10 V applied to a scanner of lateral extension of 100 µm), a maximum sliding velocity close to 6 mm/s may be achievable. Performing wear experiments at high sliding velocities also limits the drift during the experiment as it is possible to obtain a significant wear rate in a short time (Figure 2).

In our experimental set-up using a commercial AFM (DI3100 from Bruker), the diameter of the circular displacement ranges from 0.16 µm to 3 µm. Thus, the CM-AFM allows local probing of materials. In this way, it is possible to investigate wear of metallic materials while avoiding the grain boundary effects. Considering the CM-AFM, the lateral force signal (also referred as the lateral force microscopy (LFM) signal proportional to the friction force) of the cantilever stemming from the circular displacement of the contact features a sinusoidal signal with the same frequency as the relative circular displacement of the contact. Then a lock-in amplifier is used to register the real-time amplitude of the LFM signal of the cantilever (or friction force) during the wear experiment in order to determine prospective variations of the friction properties during the experiment. For example, this option may be advantageously implemented for investigating the correlation between friction, energy dissipation and worn material at the local scale or to exhibit local heterogeneity of the material.

To summarize, CM-AFM applied to wear experiments regarding different applications such as tribochemistry and wear of thin films presents numerous advantages compared to conventional AFM wear experiments: (1) sliding velocity values are much higher than the conventional ones, thus achieving a significant wear volume in a short time; (2) the drift of the piezoelectric scanner is reduced because of the short experiment duration (due to the high scanning velocity), together with average *x*- and *y*-voltage values applied on the scanner being zeroed; (3) the wear volume can be easily computed as the wear track is well-defined; (4) as the wear track is circular, it can be easily detected and unlikely to be confused with surface scratches; (5) another valuable consequence of the circular motion is that the probe, if worn during the experiment, is submitted to an isotropic wear; (6) the circular displacement allows for the exploration of wear in all directions of the surface material, which may be of major interest to investigate wear anisotropy; and (7) if wear debris are generated during the wear process, the circular motion of the tip favors rejection of this debris to the outer part of the wear track or at the exact position where this wear debris was generated, leading to an easy determination of the real wear volume. In the case of a back and forth displacement, the scanning imposed on the tip used in classical AFM nanowear experiments may result in accumulation of debris located within the wear track, which cannot be discerned from non-worn material.

### Wear volume calculation at the nanoscale

**Figure 3:**
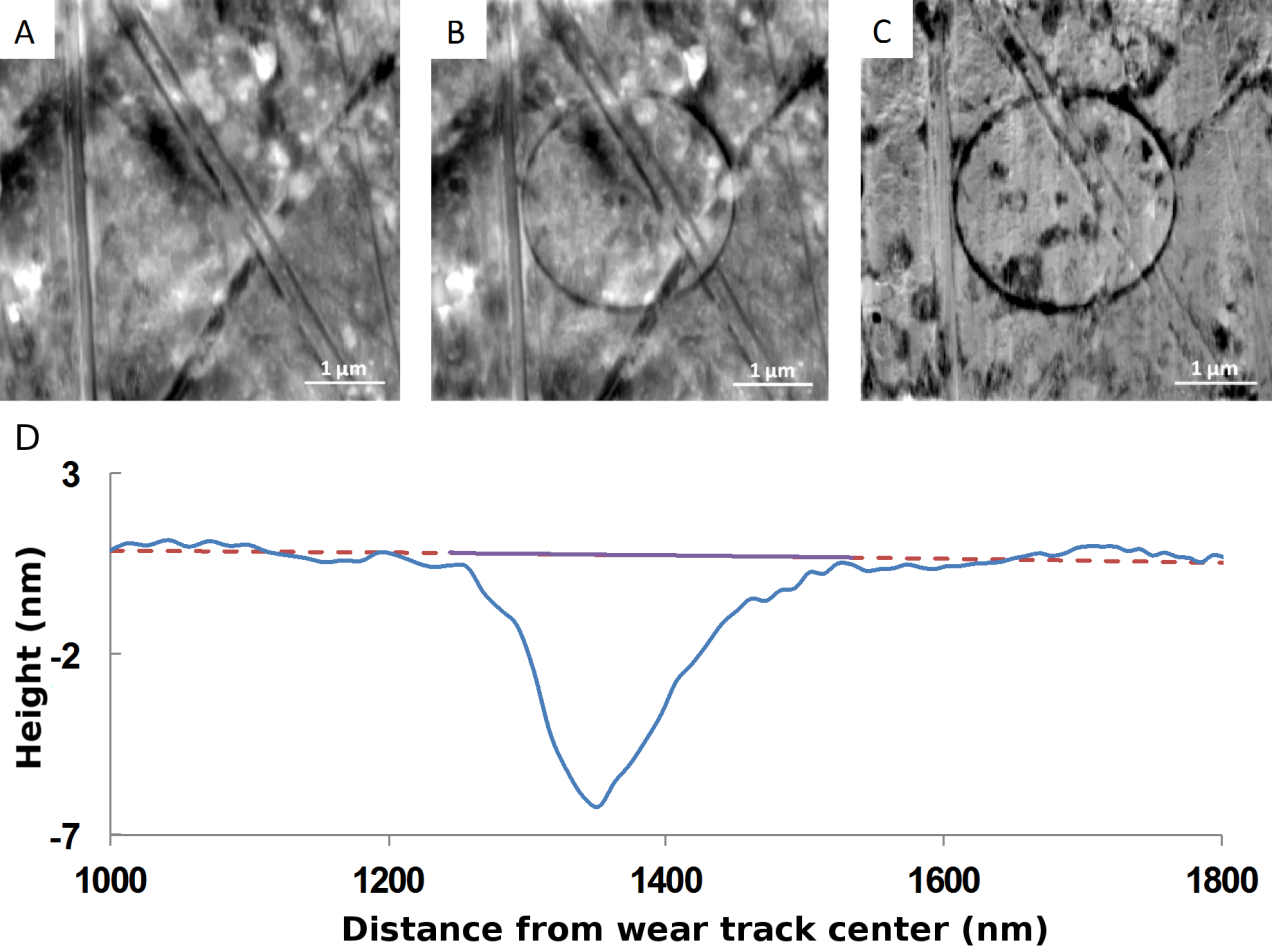
Summary of the steps for computing the wear volume from AFM topographic images (image size: 5 × 5 µm): A and B (grey scale is 35 nm) and B shows the AFM topographic images, before and after wear respectively, for a given location on a copper-based composite (the counter-body is a silicon nitride probe); C (grey scale is 16 nm) is the height difference of the topographic images (B) − (A). The drift correction has been previously determined; the bottom image D is the average height as a function of the distance from the center of the wear track circle. The baseline (dotted red line) in this example is obtained by a polynomial fit of order 4 of the profile without considering the wear track. The wear volume is calculated by integrating the surface under the full purple line.

**Figure 4:**
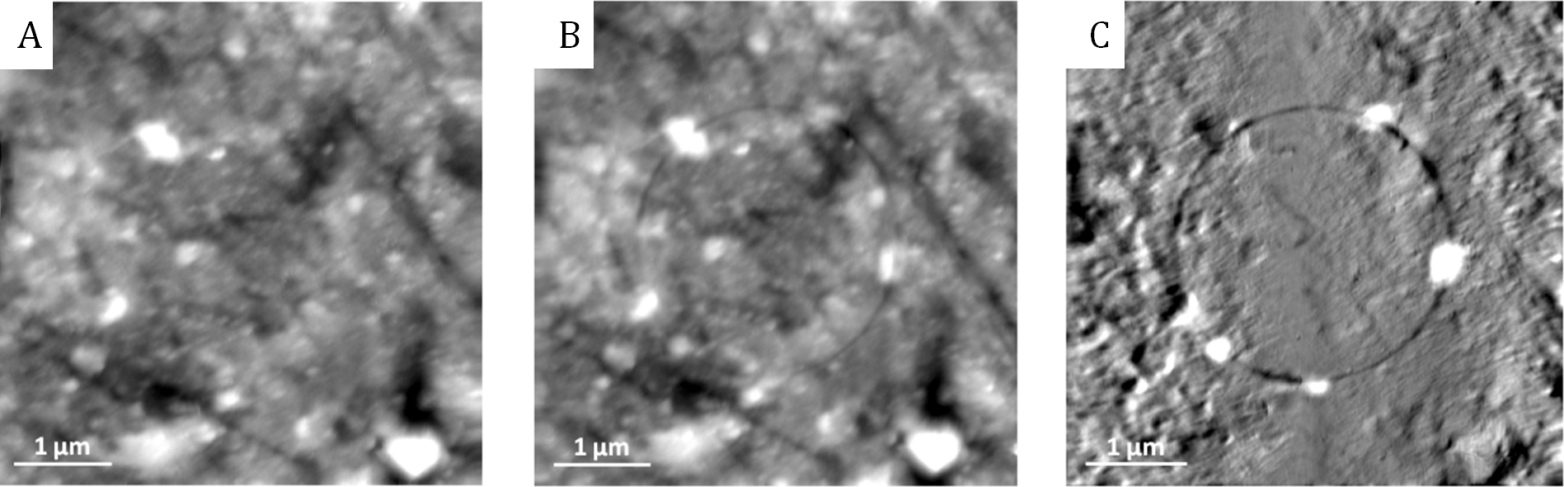
A and B (grey scale is 35 nm) are the AFM contact mode topographic images before and after the wear experiment; C (grey scale is 12 nm) is the height difference (B) − (A) obtained on a copper-based composite (image size: 5 × 5 µm).

**Figure 5:**
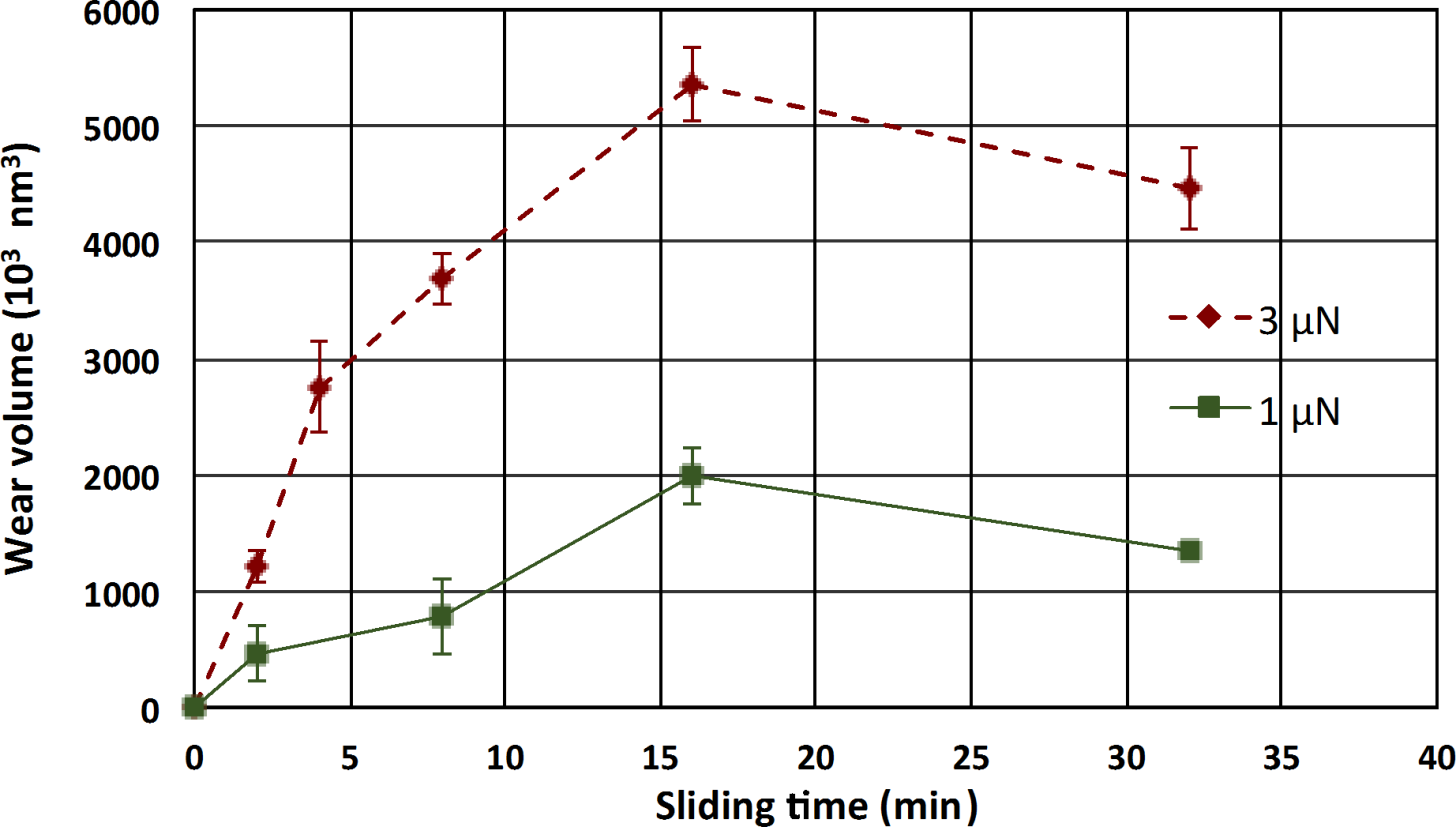
Wear volume as a function of the sliding time at a sliding velocity of 880 µm/s and a normal load of 1 µN (green squares) and 3 µN (red diamonds). Experiments were carried with a diamond-like carbon (DLC) probe. Error bars are the standard deviation and the data result from an average between the maximum wear volume and the minimum wear volume issued from the calculation described in the Experimental section.

The wear volume is calculated following a five-step procedure: 1) topographic AFM images are recorded in contact mode before and after the wear experiment, at the same location of the sample (Figure 3A,B); 2) as the wear track depth is of the same order of magnitude as the peak-to-valley roughness, it is necessary to subtract the height of these two AFM images to highlight the wear track; 3) relevant subtraction of the images must take into account the drift that occurs during the wear experiments. Therefore, both images are shifted by a few pixels in the two directions of the plane of the image and the standard deviation of the height of the pixels is computed. An optimized shift for eliminating the drift should lead to a standard deviation of the height of the pixels of the difference image converging to zero. Due to the nonconstant value of the drift along an image, the nonlinearity of the piezoelectric actuator, and the pixel size, the shifting imaging process will not be perfect. The resulting image is not perfectly flat outside the wear track, but the process considerably reduces the effect of the roughness for determining the wear volume (Figure 3C); 4) once the difference image is computed, the average height is calculated as a function of the distance from the center of the circular wear track (Figure 3D); 5) the baseline (red line) is determined by fitting the portion of the profile excluding the wear track with polynomial algorithms of order 1 to 4. The difference in height between the experimental and the four different fitted curves is integrated to compute the volume of the wear track. The minimum and maximum wear volume values of these different results are determined in order to estimate the uncertainties.

The image processing is fundamental for obtaining a clear description of the wear process. It is clearly illustrated in Figure 4 where the wear track on the image before the image processing (image B in Figure 4) is hardly distinguishable from the background. After having applied the image processing (image C), the circular wear track clearly contrasts with the background and details such as wear debris accumulation (not visible on the topographic image A before the wear experiment) become visible and help to understand the wear process.

### Application of CM-AFM to study wear of a Cu-based nanocomposite

An application example to assess the promising potential of CM-AFM to explore wear of materials at the nanoscale is addressed with a copper-based nanocomposite (see the Experimental section for details). This material is of industrial relevance as it is widely used in electrical sliding contacts such as those in railway overhead current collection system, lead frames in large-scale integrated circuitd, welding electrodes, transfer switches and electrical contact material.

Experimental details about the wear experiments with CM-AFM and the recording of the topographic images are described in the Experimental section. In particular, all wear experiments are carried out at different locations on the sample and tips of different nature were used to access to a broad range of cantilever stiffness. Experimental images as shown in Figure 3C and in Figure 4 also show that the material is not uniformly worn along the circular wear track. Wear is more intensive at some random locations of the material, evidencing heterogeneous wear (as in Figure 3C) or production of wear debris accumulation (as in Figure 4). This makes clear that CM-AFM is able to locally analyze wear mechanisms and wear properties of the material.

Figure 5 reports the evolution of the wear volume against sliding time with a given sliding velocity (880 µm/s) and two different applied normal loads, respectively 1 µN and 3 µN.

This result shows that the wear volume varies linearly with the sliding distance (running-in regime) until reaching 16 min of wear, where a plateau (steady-state regime) indicates that the wear volume is almost independent of the sliding time. This trend is similar for both values of the applied load (1 µN and 3 µN). Wear profiles (Figure 3D) obtained with the analysis procedure show also that the AFM tip conforms to the nanocomposite wear track during the wear process. The plateau could not be attributed to the wear of the probe during the wear process. At least, if the tip wear occurs, its worn volume contribution is negligible compared to the sample worn volume. One should rather consider that while the probe is going deeper and deeper into the wear track, the contact pressure is decreasing as the surface contact between the AFM tip and the substrate is increased. Consequently, the shear stress applied to the contact appears to be not high enough to wear the material.

The wear depths determined from the wear profiles (Figure 3D for example) are in the nanometer range. One can calculate from Figure 5 in the steady-state regime for an applied load of 3 µN that about 100 atoms of copper per micrometer of sliding have been removed. Then an atom-by-atom or atomic cluster by atomic cluster or nanograin-by-nanograin removal process may be involved in the wear mechanism. However, SEM imaging was of satisfactory resolution to precisely distinguish the morphology of the wear track and to confirm any of these assumptions.

**Figure 6:**
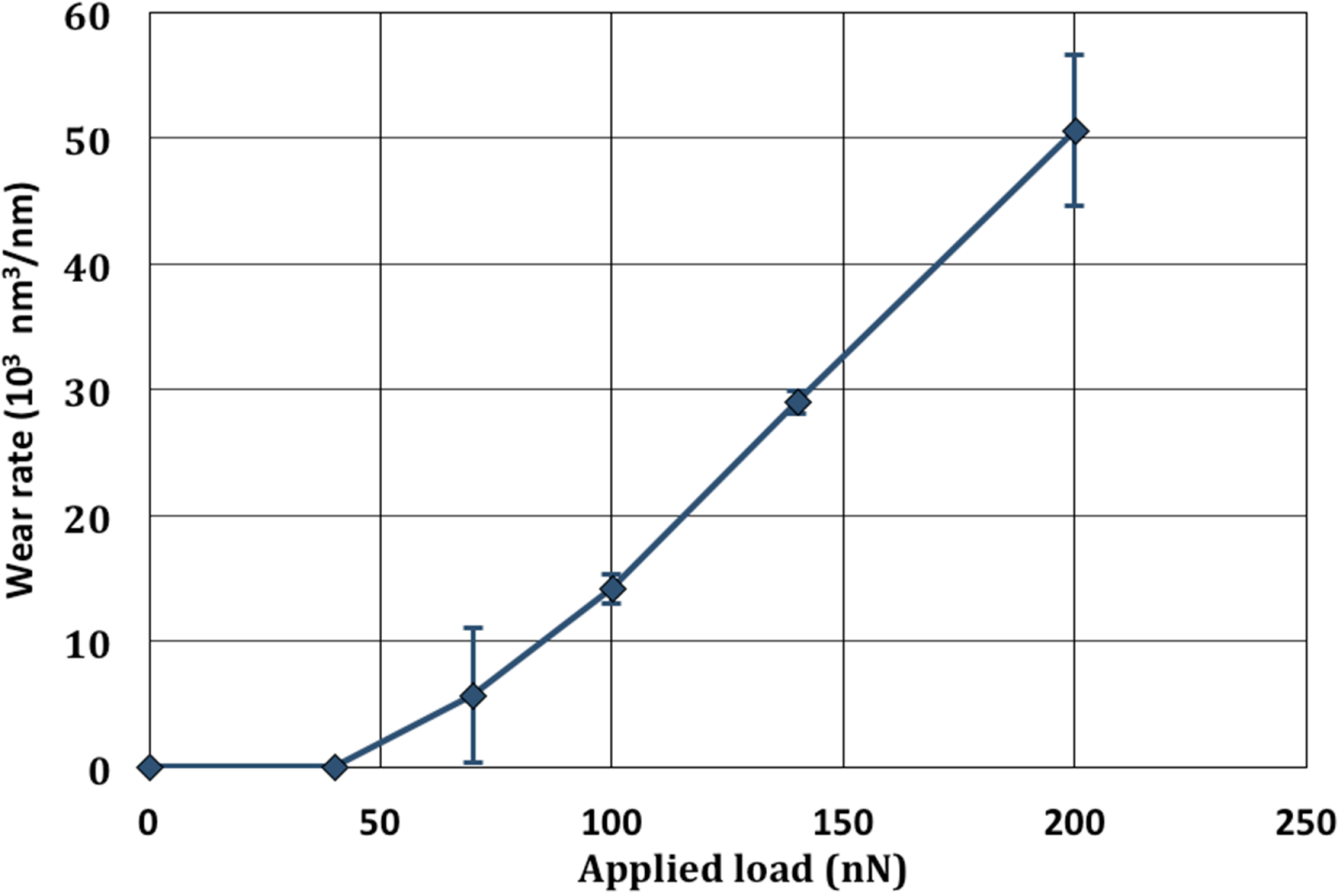
Wear rate as a function of the applied normal load with a given sliding velocity (880 µm/s) and a 2 min duration wear. Experiments were carried with the Si_3_N_4_ probe.

In the running-in regime, the evolution of the wear rate as a function of the applied normal load is depicted in Figure 6.

These results show that for high loads, the behavior follows an Archard-like wear law since the wear volume or wear rate is proportional to the applied load. For small applied loads, the wear rate is almost zero and it is necessary to reach a threshold load (of approximately 50 nN) to get a significant wear volume.

## Conclusion

This paper reported on an original approach for generating and quantitatively measuring nanoscale wear tracks resulting from the sliding contact between an AFM tip and an interacting sample (counter-body). Using the AFM circular mode with a high sliding velocity allows a significant and valuable decrease of the drift together with a well-defined wear track and consequent wear. Accordingly, quantitative wear volumes at the nanoscale are accessible using an image processing method. The used strategy to calculate the wear volume consists of attenuating the roughness of the surface that is of the same order as the nanometer dimension of the wear track (which may interfere in the calculation of the wear volume) by subtracting relevant topographic images after and before wear. Finally, this methodology has been applied to a copper-based nanocomposite sample using both diamond-like carbon and silicon nitride tips in the µN load range. The results show that even if wear loss remains locally heterogeneous and erratic, their related trends regarding Cu/Al_2_O_3_ nanocomposites at the nanoscale may be obtained.

## Experimental

The investigated copper-based nanocomposite was produced by powder metallurgy technology and contained approximately 4.7 wt % of nanometer-sized Al_2_O_3_ particles (the average diameter was less than 100 nm) [26]. The surface roughness, *R*_a_, of the samples, determined from AFM topographic images of 5 × 5 µm, is 2.9 ± 0.6 nm.

Wear experiments were performed with the CM-AFM mode implemented on a DI-3100 Nanoscope V controller Bruker AFM, in air, at ambient temperature and at a relative humidity of 30%. The AFM tips were either a unique DLC coating tip with a rectangular cantilever (from NT-MDT) or a unique silicon nitride (Si_3_N_4_) probe with a triangular cantilever. Both nominal tip radius values were 100 nm and 70 nm for the DLC and Si_3_N_4_ probes, respectively, and the cantilever stiffness was 12 N/m and 0.4 N/m, respectively (as determined by the thermal noise method [27–28]). For each set of wear experiments, a unique AFM tip was used. After each measurement, force curves on a silicon wafer were performed to verify the state of the tip. Every data point represented in Figure 5 and Figure 6 was obtained by doing wear measurements at different locations on the sample. Scanning electronic microscopy images of the probes after wear showed that no significant wear of the AFM tip occurred after the set of experiments. Such facts were also confirmed by the adhesion force measurements before each wear experiment, with quite similar resulting values. Another piece of evidence highlighting that the probe is not damaged is given by the AFM topographic images before and after each experiment, which were recorded with the same AFM tip that was used for wear experiments. In case of damaged tips, the features of the surface would have been dilated due to tip self-imaging [19]. For improving the imaging processing, AFM topographic images before and after wear were always recorded from the left to the right and from top to bottom directions. Proceeding in such a way reduces the nonlinearity behavior of the piezoelectric actuator with regards to imaging.
